# Supplementary material for: SARS-CoV-2 Nsp1 suppresses host but not viral translation through a bipartite mechanism
Source: bioRxiv. 2020 Sep 18:2020.09.18.302901. Preprint. [Version 1] doi: 10.1101/2020.09.18.302901 (PMC7523103; doi:10.1101/2020.09.18.302901)
Supplement: 1 [file NIHPP2020.09.18.302901-supplement-1.pdf]

bioRxiv preprint doi: https://doi.org/10.1101/2020.09.18.302901. this version posted September 20, 2020. The copyright holder for this preprint (which was not certified by peer review) is the author/funder. It is made available under a CC-BY 4.0 International license.

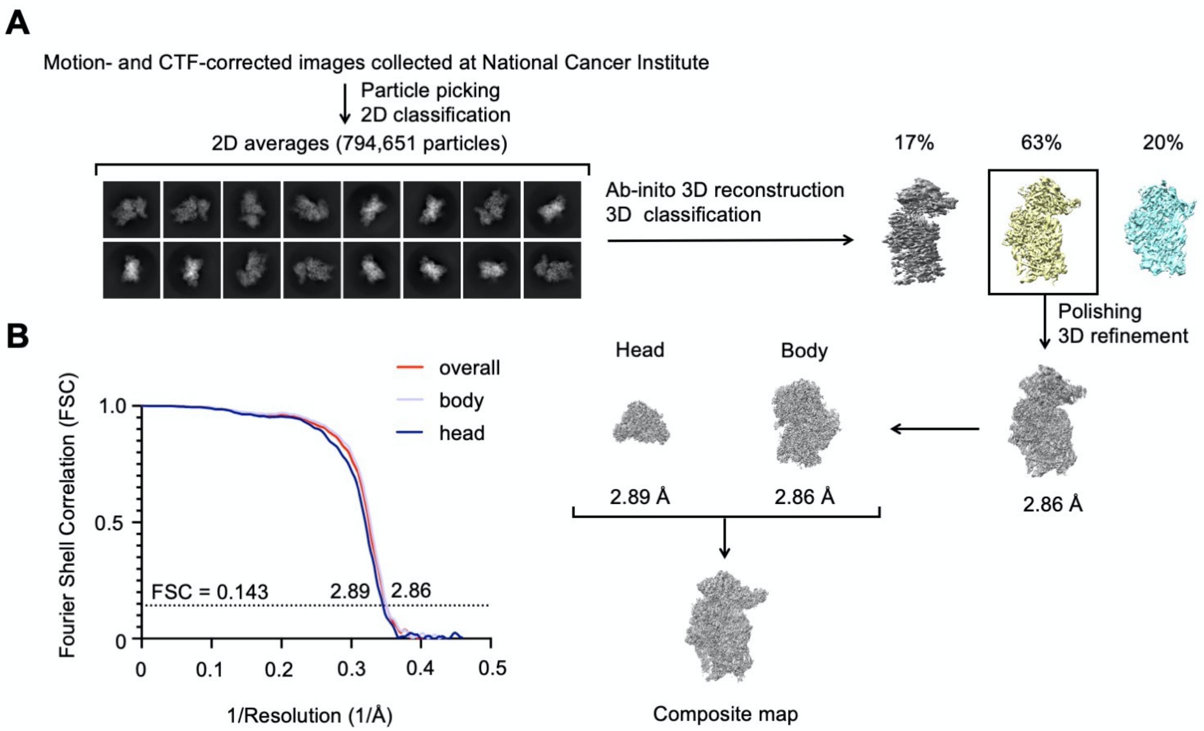

Figure S1. Reconstruction of SARS-CoV-2 Nsp1 bound to human 40S ribosomal subunit.
(A) Workflow of 3D reconstruction. The selected map is boxed in rectangles.
(B) Fourier shell correlation (FSC) curves of 3D reconstructed complex of SARS-CoV-2 Nsp1 and human 40S ribosomal subunit.
